# Genetic diversity and variation of seven Chinese grass shrimp (*Palaemonetes sinensis*) populations based on the mitochondrial *COI* gene

**DOI:** 10.1186/s12862-021-01893-8

**Published:** 2021-09-06

**Authors:** Yingying Zhao, Xiaochen Zhu, Ye Jiang, Zhi Li, Xin Li, Weibin Xu, Hua Wei, Yingdong Li, Xiaodong Li

**Affiliations:** 1 Key Laboratory of Zoonosis of Liaoning Province, College of Animal Science and Veterinary Medicine, Shenyang Agricultural University, Shenyang, 110866 China; 2 College of Science and Engineering, Flinders University, Bedford Park, SA 5024 Australia; 3 Panjin Guanghe Crab Industry Co. Ltd., Panjin, 124000 China

**Keywords:** *Palaemonetes sinensis*, *COI* gene, Genetic variability, Population genetics

## Abstract

**Background:**

Chinese grass shrimp (*Palaemonetes sinensis*) is an important species widely distributed throughout China, which is ecologically relevant and possesses ornamental and economic value. These organisms have experienced a sharp decline in population due to overfishing. Therefore interest in *P. sinensis* aquaculture has risen in an effort to alleviate fishing pressure on wild populations. Therefore, we investigated the genetic diversity and variation of *P. sinensis* to verify the accuracy of previous research results, as well as to assess the risk of diversity decline in wild populations and provide data for artificial breeding.

**Methods:**

*Palaemonetes sinensis* specimens from seven locations were collected and their genetic variability was assessed based on mitochondrial *COI* gene segments. DNA sequence polymorphisms for each population were estimated using DNASP 6.12. The demographic history and genetic variation were evaluated using Arlequin 3.11. At last, the pairwise genetic distance (*Ds*) values and dendrograms were constructed with the MEGA 11 software package.

**Results:**

Our study obtained sequences from 325 individuals, and 41 haplotypes were identified among the populations. The haplotype diversity (*H*_*d*_) and nucleotide diversity (*π*) indices ranged from 0.244 ± 0.083 to 0.790 ± 0.048 and from 0.0004 ± 0.0001 to 0.0028 ± 0.0006, respectively. Haplotype network analyses identified haplotype Hap_1 as a potential maternal ancestral haplotype for the studied populations. AMOVA results indicated that genetic variations mainly occurred within populations (73.07%). Moreover, according to the maximum variation among groups (*F*_*CT*_), analysis of molecular variance using the optimal two-group scheme indicated that the maximum variation occurred among groups (53.36%). Neutrality and mismatch distribution tests suggested that *P. sinensis* underwent a recent population expansion. Consistent with the SAMOVA analysis and haplotype network analyses, the *D*s and *F*_*ST*_ between the population pairs indicated that the JN population was distinctive from the others.

**Conclusions:**

Our study conducted a comprehensive characterization of seven wild *P. sinensis* populations, and our findings elucidated highly significant differences within populations. The JN population was differentiated from the other six populations, as a result of long-term geographical separation. Overall, the present study provided a valuable basis for the management of genetic resources and a better understanding of the ecology and evolution of this species.

**Supplementary Information:**

The online version contains supplementary material available at 10.1186/s12862-021-01893-8.

## Background

Chinese grass shrimp (*Palaemonetes sinensis*) is an important species belonging to the Palaemonidae family, which is widely distributed throughout China [20, 23]. These organisms are not only ecologically relevant but also possess ornamental and economic value [16, 36]. Additionally, *P. sinensis* is very popular in both domestic and foreign markets due to its pleasant flavor and high nutritional value [16, 37]. However, the wild populations of *P. sinensis* have gradually declined due to environmental pollution and overharvesting, and therefore interest in *P. sinensis* aquaculture has risen in an effort to potentially alleviate fishing pressure on wild populations by producing farmed shrimp to meet consumer demand. Therefore, our research group has conducted several studies on *P. sinensis*, including studies on morphology [38], physiology [3], immunology [9, 21, 22], genetic diversity [36], and phylogenetic relationships [37] among others. However, studies on the population genetic diversity and structure of this species remain scarce. It is well known that the study of population genetic diversity and genetic structure could provide guidance for the establishment of fishing quotas to prevent overharvesting [36]. At the same time, it is very necessary to understand the genetic structure and genetic diversity of wild *P. sinensis* populations, and to correctly evaluate the status of germplasm resources prior to artificial breeding, which can provide a basis for the selection of breeding populations [36]. Therefore, more research is needed to understand the genetics of this species.

*COI* gene sequence polymorphisms serve as “barcodes” to identify different species and assess cryptic diversity [15, 31] and have thus been increasingly used to investigate population genetics, taxonomy, molecular evolution, phylogeny relationship origins, and the diversity of Palaemonidae species such as *Macrobrachium australiense* [4], *Macrobrachium olfersii* [25], *Palaemon longirostris* and *Palaemon garciacidi* [5], and *Palaemon capensis* and *Palaemon peringueyi* [32]. In our previous study, we investigated the genetic diversity and structure of *P. sinensis* using transcriptome-derived microsatellite markers [37]. The results indicated that two populations, LD and SJ, had the lowest genetic diversity and were markedly different from the other populations. Given that microsatellites represent changes in the nuclear genome, the microsatellite markers obtained via transcriptome analysis represented sequences of coding regions in the genome, which are highly conserved and may underestimate population genetic diversity. Therefore, further studies are needed to establish the relationship and genetic variability among *P. sinensis* populations in China using different molecular markers. The *COI* gene polymorphisms represent changes in the mitochondrial DNA (mtDNA) genome. Thus, in this study, *COI* gene fragments were characterized to investigate the genetic diversity and population structure of seven wild *P. sinensis* populations in China. Through this study, we sought to verify the accuracy of previous research results, assess whether there is a risk of diversity decline in wild populations, and provide data for artificial breeding. Additionally, this genetic survey provides valuable information for the development of effective conservation and management strategies for this species and establishes a theoretical basis for the future study of the genetic biodiversity of *P. sinensis*.

## Methods

### Sampling, DNA extraction, and sequencing

**Fig. 1:**
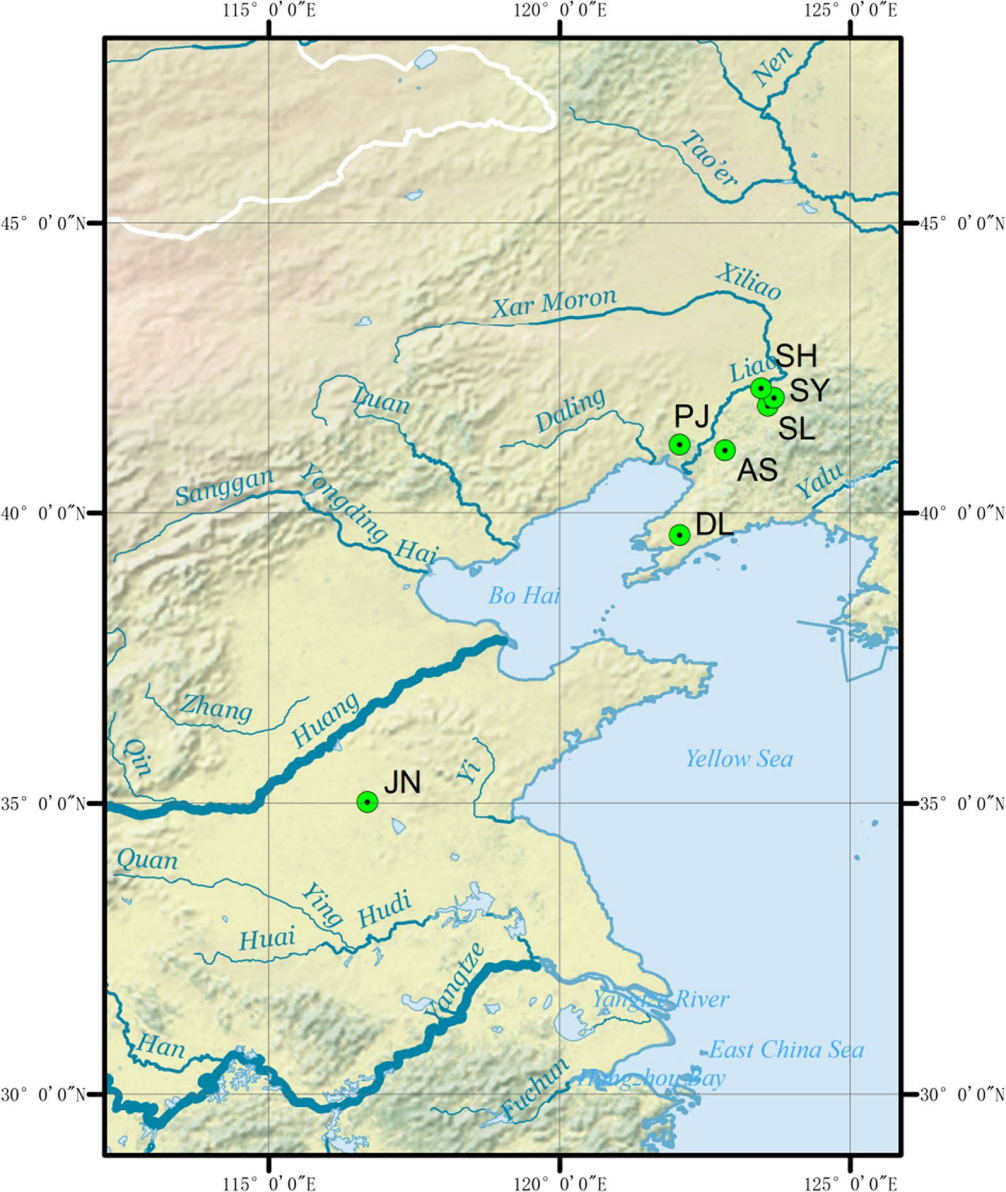
Sampling collection sites of *P. sinensis* (see Table 1 for the full description of the populations). ArcMap 10.4 was used to produce a distribution map. The base map for the depiction was obtained freely from the URL: https://www.naturalearthdata.com/downloads/

A total of 326 wild individuals were collected from seven locations in China (Table 1, Fig. 1), and stored in 75% alcohol directly. Genomic DNA was extracted from individual muscle samples using the TIAnamp Marine Animals DNA Kit (TIANGEN, China) according to the manufacturer’s instructions, and the quality of the extracted DNA was assessed via electrophoresis on a 1% agarose gel coupled with spectrophotometric analyses using a Thermo Scientific NanoDrop 2000 system.

**Table 1:** Sampling localities, geographic position of *P. sinensis*

Populations	Sampling locations	Geographic position		Sample size
DL	Liaoning Dalian Sha River	39.622° N	122.067° E	46
PJ	Liaoning Panjin Shuangtaizi River	41.180° N	122.067° E	44
AS	Liaoning Anshan Yangliu River	41.082° N	122.847° E	47
SL	Liaoning Shenyang Longwei Lake	41.842° N	123.589° E	47
SY	Liaoning Shenyang Yangshi reservoir	41.978° N	123.691° E	48
SH	Liaoning Shenyang Huangjia Liao River	42.146° N	123.472° E	48
JN	Shandong Jining Dushan Lake	35.033° N	116.702° E	46

The *COI* gene sequence was partially amplified via polymerase chain reaction (PCR) using the LCO1490 and HCO2198 primers [13]. Each PCR reaction (25 μL per reaction) contained 100 ng of template, forward and reverse primers (10 μmol/L; 1 μL each), 12.5 μL of 2× *Taq* PCR Master Mix (TIANGEN, China), and ddH_2_O [35]. Denaturation was conducted for 3 min at 94 ℃, followed by 35 cycles at 94 ℃ for 30 s, 53 ℃ for 40 s, and 72 ℃ for 40 s, with a final prolonged extension step at 72 ℃ for 10 min. The PCR products were then assessed via electrophoresis in 1% agarose gels [35], then purified and sequenced by Sangon Biotech (Shanghai) Co., Ltd.

### Data analysis and phylogenetic relationship

All *COI* gene fragments were sequenced in both directions and the assembled sequences were manually inspected for quality assurance. Afterward, all sequences were aligned using Clustal Omega (https://www.ebi.ac.uk/Tools/msa/clustalo/) [28]. DNA sequence polymorphisms including the highest number of haplotypes (*h*), number of segregating sites (*S*), average number of differences (*K*), haplotype diversity (*H*_*d*_), and nucleotide diversity (*π*) values for each population were estimated using DNASP 6.12 [26]. Haplotypes for specific sample populations were also identified with DNASP 6.12 and haplotype networks were visualized with the PopART (Population Analysis with Reticulate Trees) network analysis software [19] using the TCS network inference method [8].

To estimate demographic history, Tajima’s *D* and Fu’s *Fs* tests were used to examine neutrality, and mismatch distribution analysis was also performed to assess the population expansion hypothesis using Arlequin 3.11 (10,000 permutations) [11]. Additionally, the relative population sizes before (*θ*_*0*_) and after (*θ*_*1*_) were also determined using Arlequin 3.11. The correlation between the observed and simulated distributions was tested using the SSD and HRI. The time of expansion was calculated with the formula

$${\text{T}} =\uptau /{2}\upmu ,$$

where μ = generation time × number of base pairs per sequence × mutation rate for the marker used, and τ was calculated in Arlequin 3.11. A mutation rate of 1.4% per million years [18] and a generation time of 1.5 years were assumed for all calculations. This generation time was established based on previous literature on other Palaemonidae shrimps species [32].

Genetic variation was evaluated via AMOVA [12] and pairwise *F*_*ST*_ were calculated using Arlequin 3.11 with 10,000 permutations [11]. SAMOVA 2.0 [10] was used to identify the defined drainage groups in the greatest variation among groups (*F*_*CT*_). The *Ds* values among populations based on the Kimura 2-parameter model were then calculated, after which the UPGMA and NJ dendrograms were constructed with the MEGA 11 software package [29].

## Results

### Sequence variation and genetic diversity

674 base pair sequences of the *COI* gene was obtained for downstream analyses. In 326 individuals of seven populations, 37 polymorphic sites were identified in this sequence, 21 of which were parsimony-informative. Moreover, the highest number of *h*, *S*, *K*, *H*_*d*_, and *π* values were observed in the SL population (*h* = 15, *S* = 25, *K* = 1.885, *H*_*d*_ = 0.790 ± 0.048, and *π* = 0.0028 ± 0.0006), whereas the lowest values were observed in the DL population (*h* = 5, *S* = 4, *K* = 0.257, *H*_*d*_ = 0.244 ± 0.083, and *π* = 0.0004 ± 0.0001) (Table 2). Overall, most locations exhibited moderate to high haplotype diversity (0.244–0.790) due to large number of unique haplotypes. However, nucleotide diversity was relatively low, ranging from 0.0004 to 0.0028.

**Table 2:** List of the genetic diversity estimates of *P. sinensis* populations

Population	*h*	*h*ʹ	*S*	*K*	*H*_*d*_ ± SD	*π* ± SD
DL	5	3	4	0.257	0.244 ± 0.083	0.0004 ± 0.0001
PJ	10	6	12	0.884	0.371 ± 0.094	0.0013 ± 0.0005
AS	6	2	8	0.650	0.309 ± 0.085	0.0010 ± 0.0004
SL	15	10	25	1.885	0.790 ± 0.048	0.0028 ± 0.0006
SY	8	5	6	0.691	0.539 ± 0.071	0.0010 ± 0.0002
SH	6	2	8	0.959	0.601 ± 0.070	0.0014 ± 0.0004
JN	7	5	9	0.588	0.382 ± 0.088	0.0009 ± 0.0003
Mean				1.116	0.627 ± 0.029	0.0017 ± 0.0005

*h*: Number of haplotypes; *h*ʹ: No. of unique haplotypes; *S*: Number of segregating sites; *K*: Average number of differences; *H*_*d*_: Haplotype diversity; *π*: Nucleotide diversity; SD: standard deviation

### Haplotype network analysis

**Fig. 2:**
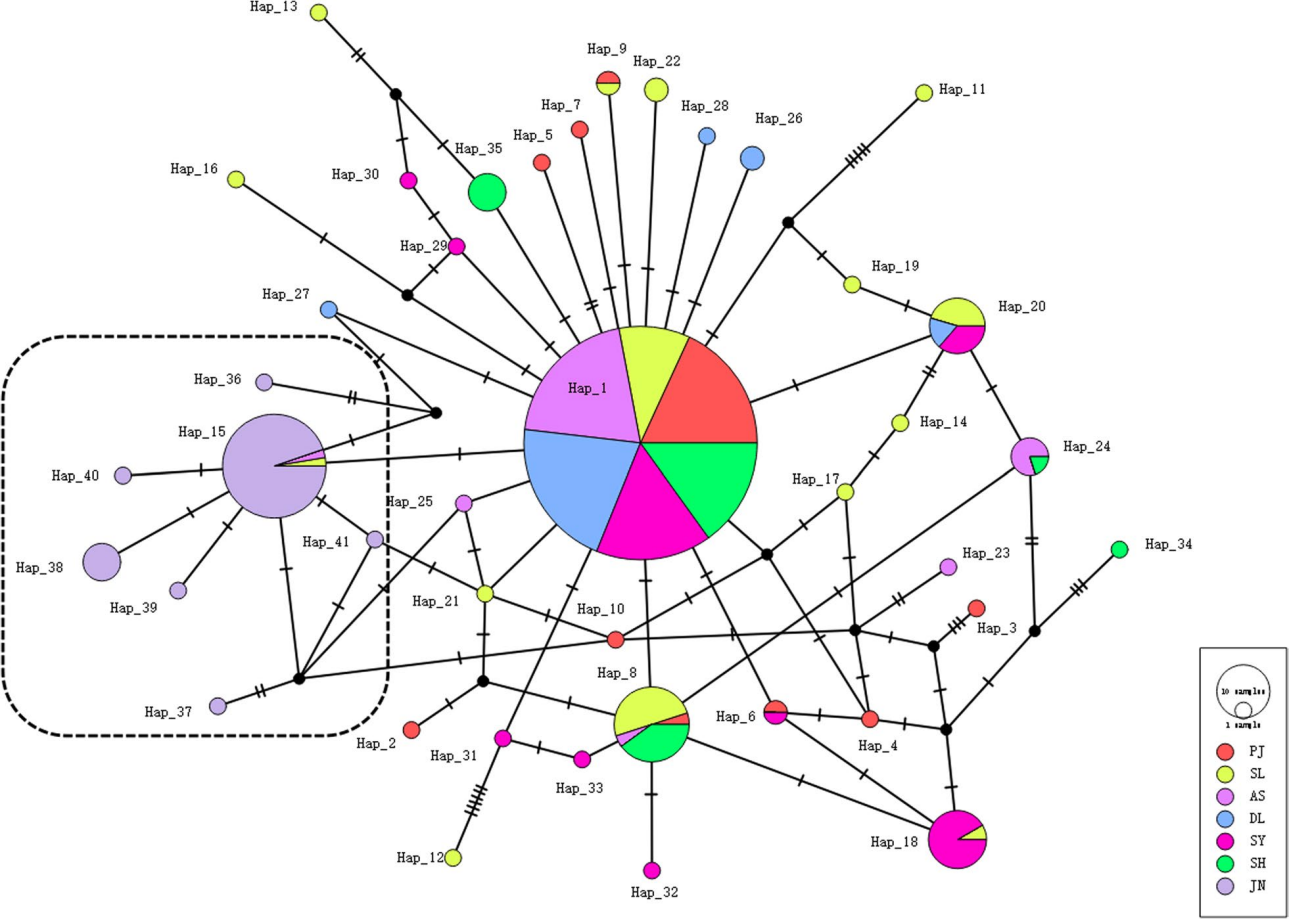
TCS network of *P. sinensis* based on *COI* haplotypes. The sizes of the circles indicate the number of individuals with each given haplotype. The number in the bracket represents the mutation steps between haplotypes and the black circles on the line represent an additional mutational change

A total of 41 haplotypes were defined according to all variable positions in the *COI* gene from seven populations (Additional file 1: Table S1, GenBank Accession numbers: MT884019–MT884059). Among these haplotypes, 29 were singleton. For the shared haplotypes, 193 out of 326 (59.20%) individuals belong to one haplotype (Hap_1) in six populations (excluding JN population). There were 6 to 15 unique haplotypes within the populations but only eight haplotypes were shared between/among populations and no common haplotype was shared across all populations. A genealogy with one essential haplotype (Hap_1) was identified from the haplotype network based on the TCS inference method and all other haplotypes arose from it through one or several mutational steps (Fig. 2). Due to its central position in the network and exhibiting the highest frequency, haplotype Hap_1 might be the maternal ancestral haplotype for the *P. sinensis* populations in this study. Among other haplotypes, seven shared haplotypes were identified (Hap_6, Hap_8, Hap_9, Hap_15, Hap_18, Hap_20, and Hap_24) and 33 unique haplotypes were found to derive from the above-mentioned haplotypes within seven mutational steps. For the JN population, six singleton haplotypes were derived from its dominant haplotype (Hap_15) within one or two mutation steps.

### Population expansion

Both Tajima’s *D* and Fu’s *Fs* tests showed negative values for all populations (Table 3), most of which reached a significant level (*P* < 0.05). The results for four populations (DL, PJ, SL, and JN) were found to be extremely significant (*P* < 0.01) for both tests, indicating a departure from mutation-drift equilibrium and potential population demographic expansion.

**Table 3:** Neutrality test and mismatch distribution analysis of *P. sinensis* populations

Population	Neutrality test		Mismatch distribution analysis					Expansion time
	Tajima *D*	*Fu’s Fs*	τ	*θ*_*0*_	*θ*_*1*_	SSD	HRI	*t*(Ma)
DL	− 1.659*	− 4.070**	3.000	0.000	0.341	0.003	0.322	0.106
PJ	− 2.057**	− 6.494**	3.000	0.000	0.571	0.010	0.240	0.106
AS	− 1.928*	− 2.448	3.000	0.000	0.421	0.018	0.359	0.106
SL	− 2.201**	− 7.914**	1.387	0.000	99,999.000	0.007	0.070	0.049
SY	− 1.254	− 4.723*	0.768	0.000	99,999.000	0.003	0.098	0.027
SH	− 1.287	− 1.207	0.861	0.000	99,999.000	0.013	0.130	0.030
JN	− 2.153**	− 4.119**	0.469	0.000	9.455	0.007	0.190	0.017
mean	− 1.751*	− 4.425*	1.783	0.000	42,858.255	0.009	0.201	0.063

τ: age of expansion in units of mutational time; *θ*_*0*_: population size before expansion; *θ*_*1*_: population size after expansion; SSD: sum of squared deviations; HRI: Harpending’s raggedness index; *t*: Expansion time; Ma: millions of years ago

*P < 0.05; **P < 0.01

**Fig. 3:**
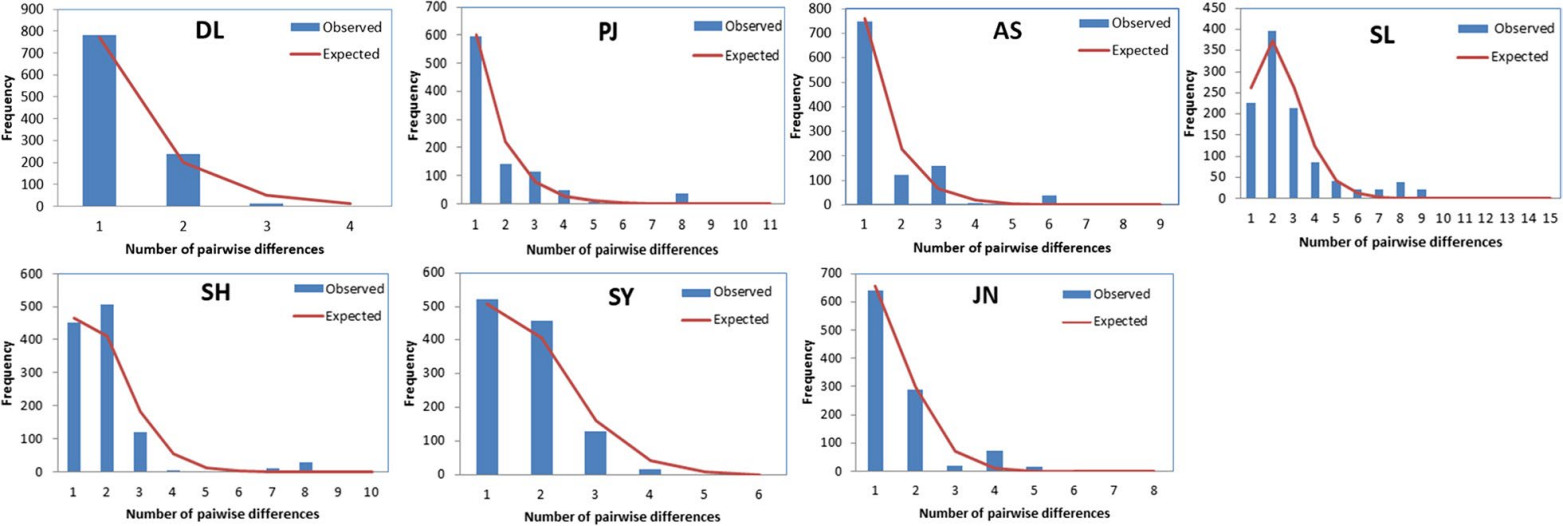
Mismatch distributions of the *P. sinensis* haplotypes in each population. The bars represent the observed values, whereas the curves represent the expected values

The population sizes before expansion (*θ*_*0*_) were zero for all populations, whereas population size after expansion (*θ*_*1*_) in the SL, SY, and SH populations were noticeably larger than in the other populations (Table 3). Furthermore, mismatch distribution did not differ significantly from the sudden expansion model when using either the sum of squared deviations (SSD) or Harpending’s raggedness index (HRI) for goodness-of-fit. A pattern of population expansion for *P. sinensis* was supported by the unimodal mismatch analysis (Fig. 3). Further, SSD and HRI were not statistically different from the model-predicted frequency (SSD = 0.009, *P* > 0.05; HRI = 0.201, *P* > 0.05) (Table 3).

Coalescence analyses demonstrated that the age of expansion in units of mutational time (τ) was highly variable (0.469–3.000) among the populations. Using a mutation rate of 1.4% per million years [18], the expansion of the *P. sinensis* population was estimated to have occurred approximately 16,000 to 106,000 years ago. The value of τ for the entire dataset was 1.783, corresponding to a demographic expansion origin of approximately 63,000 years before the present time for *P. sinensis* (Table 3).

### Genetic divergence and distance among populations

The analysis of molecular variance (AMOVA) test indicated that the genetic variances within and among populations were 26.93% and 73.07%, respectively (Table 4). Furthermore, separating the seven populations into two groups maximized the variation among groups (*F*_*CT*_) as suggested by spatial analysis of molecular variance (SAMOVA) analysis (Table 4). The two groups were PJ + SL + AS + DL + SY + SH and JN. Analysis of molecular variance using the optimal two-group scheme indicated that the highest variation (53.36%) occurred among groups, whereas the variance within populations was 45.11%.

**Table 4:** Results of AMOVA and SAMOVA of *P. sinensis* populations

	Groups	Source of variation	*d.f*	Sum of squares	Variance components	Percentage of variation (%)	Fixation indices
AMOVA	One group	Among populations	6	46.159	0.156	26.93	*F*_*ST*_ = 0.269**
		Within populations	319	135.133	0.424	73.07	
		Total	325	181.291	0.580		
SAMOVA	1. PJ + SL + AS + DL + SY + SH	Among groups	1	40.618	0.501	53.36	*F*_*SC*_ = 0.033**
	2. JN	Among populations within groups	5	5.478	0.014	1.53	*F*_*ST*_ = 0.549**
		Within populations	319	135.133	0.424	45.11	*F*_*CT*_ = 0.534
		Total	325	181.291	0.939		

*d*.*f*.: Degree of freedom

**P < 0.01

**Fig. 4:**
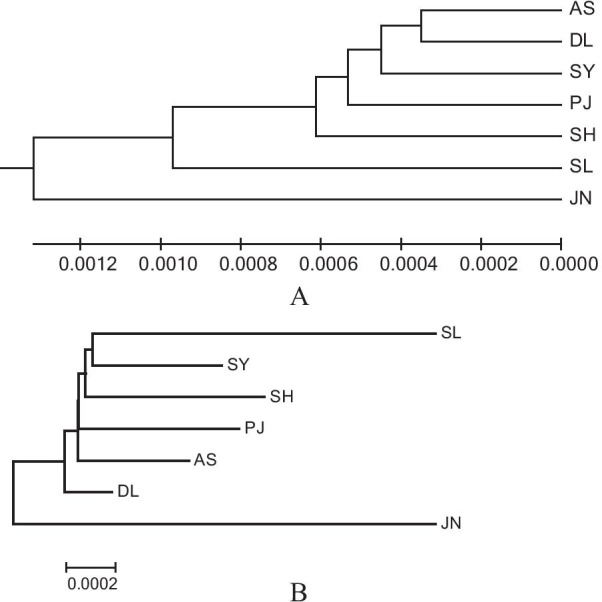
UPGMA (**A**) and NJ (**B**) clustering of *P. sinensis* populations based on the mitochondrial *COI* gene

The pairwise Wright’s fixation index (*F*_*ST*_) values among the seven studied populations ranged from − 0.0029 to 0.7052, most of which were highly significant (*P* < 0.01) (Table 5). Additionally, based on the criteria for genetic differentiation proposed herein, JN exhibited very high genetic differentiation (*F*_*ST*_ > 0.25) relative to other populations, as demonstrated by its *F*_*ST*_ values ranging from 0.4555 to 0.7052, all of which were found to be extremely significant [33]. The pairwise genetic distance (*Ds*) values between the seven examined *P. sinensis* populations were calculated according to the Kimura 2-parameter model (Table 5, below diagonal). The values ranged from 0.0007 to 0.0034, further supporting the low genetic differentiation between the populations. Figure 4 illustrates the generated UPGMA and NJ trees based on the Kimura 2-parameter genetic distance. The JN population was separated from all six remaining populations.

**Table 5:** Pairwise *F*_*ST*_ (above diagonal) and *D*_*s*_ (below diagonal) of *P. sinensis*

	PJ	SL	AS	DL	SY	SH	JN
PJ		0.0283**	0.0045	0.0250**	0.0521**	0.0407*	0.5805**
SL	0.0021		0.0079	0.0586**	0.0123	− 0.0029	0.4555**
AS	0.0011	0.0019		0.0208	0.0384*	0.0105	0.6124**
DL	0.0009	0.0017	0.0007		0.1317**	0.0790**	0.7052**
SY	0.0012	0.0019	0.0010	0.0008		0.0228	0.6277**
SH	0.0014	0.0021	0.0012	0.0010	0.0013		0.5790**
JN	0.0026	0.0034	0.0024	0.0021	0.0026	0.0027	

*P < 0.05, **P < 0.01

## Discussion

### Genetic diversity

Previous reports have explored the genetic diversity of different Palaemonidae populations based on the mtDNA *COI* gene. However, our study is the first to examine the population-wide genetic variability of *P. sinensis* using mtDNA. Compared with other Palaemonidae, the haplotype diversity (*H*_*d*_ = 0.627) of *P. sinensis* was similar to that of *P. capensis* (*H*_*d*_ = 0.607) and *P. peringueyi* (*H*_*d*_ = 0.795) in South Africa [32], but lower than several other species such as *M. australiense* (*H*_*d*_ = 0.924) [4], *M. olfersii* (*H*_*d*_ = 0.94) [25], and *P. longirostris* and *P. garciacidi* (*H*_*d*_ = 0.97) [5]. The lower genetic diversity observed herein was similar to our previous reports using microsatellite markers, which may be due to poor swimming ability, as well as the time or space constraints during *P. sinensis* sampling [37].

Overall, the *P. sinensis* populations showed high haplotype diversity (*H*_*d*_ = 0.627) but low nucleotide diversity (*π* = 0.0004) in this study. The remarkably lower nucleotide diversity (*π* < 0.005) may be due to the commercial overexploitation of wild stocks, as *P. sinensis* is a prevalent specialty food and is also sought-after as a pet in this region. During sample collection, we found that some fishermen would engage in illegal fishing during the closed season, and this illegal fishing would lead to a sharp decline in the population size, resulting in a decrease in genetic diversity. Overharvesting can drive the decline of genetic diversity [1, 24, 27, 34], which is closely related to the long-term adaptability and survivability of populations. Low genetic diversity can affect the fitness [6] and the ability of individuals to survive and adapt to future environments [27, 34]. Therefore, the unnecessary cross-basin introduction should be avoided to preserve the local genetic resources. Moreover, in addition to fishing restrictions, the population size of low genetic diversity populations can be recovered by releasing shrimp from adjacent populations.

### Genetic variation

Based on the outcome of AMOVA, the genetic differentiation within populations of *P. sinensis*, which accounted for 73.07% of the total genetic variation, was much higher than that among populations. Moreover, according to the maximum variation among groups (*F*_*CT*_) determined by SAMOVA, analysis of molecular variance using the optimal two-group scheme (JN and the other populations) indicated that the highest variations were found among groups (53.36%). Similarly, both the pairwise *Ds* and *F*_*ST*_ values revealed that the JN population was genetically different from the other populations. According to the criterion made by Thorp [30], the pairwise *Ds* values between pairs of *P. sinensis* populations ranged between 0.0007 and 0.0034, indicated that they were closely populations (*Ds* < 0.2). In contrast, the pairwise *F*_*ST*_ values among the other six populations were low or moderate [2]. The low or moderate differences between the pairwise *F*_*ST*_ values of the six populations indicated that they might share the same ancestors, and the haplotype analysis results also supported this conclusion. This was likely because the samples in the present study were collected from two geographical areas. Specifically, the JN population belonged to the Huaihe River Drainage in East China, whereas the other six populations inhabit the Liaohe River Basin and Related River Drainage in Northeast China. Therefore, the divergence between the JN population and the other six populations is due to the long-term geographical separation of their ancestors, which is consistent with our previous study using microsatellite markers [37]. Nevertheless, although no apparent geographic division was found based on genealogic reconstructions (Fig. 2) and the haplotypes of the JN population were distinct from those of the other populations, no distinct branches were observed in the Fig. 2. This may be because the estimated time of population expansion (16,000 to 106,000 years ago) was too short to form geographically unique clade [7].

### Historical demographics

Neutrality tests (Tajima’*D* and Fu’s *F*_*s*_) and mismatch distribution analysis were performed to understand the demographic dynamics of seven *P. sinensis* populations. Both mismatch distribution analysis and significant negative values of neutrality tests indicated a pattern of recent population expansion in most populations. The insignificant values of *P*_*SSD*_ and *P*_*Rag*_ (*P* > 0.05) did not reject the hypothesis of the population growth in all seven populations. Moreover, the extremely significant negative values in the majority of Fu’s *F*_*s*_ statistics in populations DL, PJ, SL, SY, and JN also confirmed the expansions of these five populations despite the negative but non-significant Fu’s *F*_*s*_ value in population AS and SH.

The demographic history was reflected in the genetic indices of this species, which showed low nucleotide diversity (*π* < 0.005) and higher haplotype diversity (*H*_*d*_ > 0.5) (Table 2). As described by Grant et al. [14], high *H*_*d*_ and low *π* values can be attributed to rapid population expansion after a period of low effective population size, which enhances the retention of new mutations. This is consistent with the large number of unique and low-frequency haplotypes found in the present study (Additional file 1: Table S1). This is also consistent with the negative Tajima’s *D* value observed in our study, which was probably due to the population expansion caused by larger scale breeding after a sharp decline in population size [17]. Additionally, low diversity parameters (*H*_*d*_ < 0.5, *π* < 0.005) were identified in the DL, PJ, AS and JN populations, indicating that they may have recently experienced a bottleneck or founder effect produced by minority populations [14]. The genetic purity of these populations was not suitable for further selective breeding as a base population. Therefore, in situ conservation and the introduction of individuals from adjacent populations is critical in order to improve genetic diversity and reduce diversity decline as a result of inbreeding [14, 17].

## Conclusions

The genetic variability of seven *P. sinensis* populations was assessed based on partial sequences of the *COI* gene. The results of AMOVA showed highly significant differences within populations. Forty-one haplotypes were detected among the populations, and the haplotype network indicated that haplotype Hap_1 might be the maternal ancestral haplotype for the studied *P. sinensis* populations. Neutrality and mismatch distribution tests suggested that *P. sinensis* underwent recent population expansion. The result of SAMOVA analysis, haplotype network analysis, *D*_*s*_, and *F*_*ST*_ among pairs of populations suggested that the JN population was distinctive from the other six populations, which was due to long-term geographic separation. Overall, our results provide important insights for the development of genetic resource management strategies and enable a better understanding of the ecology and evolution of this species. Lastly, we propose several strategies for future *P. sinensis* genetic germplasm protection and aquaculture. First, illegal fishing should be strictly limited during the closed fishing season to avoid reductions in *P. sinensis* population size. Second, the DL, PJ, AS and JN populations were not suitable for further selective breeding as a base population, which highlights the need for in situ conservation efforts. Moreover, populations with low genetic diversity can be recovered by releasing shrimp from adjacent populations, and unnecessary cross-basin introduction should be avoided to preserve the local genetic resources.

## Supplementary Information

**Additional file 1: Table S1.** Distribution of the *COI* haplotypes of *P. sinensis*.

## Data Availability

Sequence data from this article have been deposited with the GenBank Data Library under the Accession numbers: MT884019–MT884059.

## Abbreviations

COI: Cytochrome *c* oxidase subunit I
mtDNA: Mitochondrial DNA
DNA: Deoxyribonucleic acid
*h*: Number of haplotypes
*S*: Number of segregating sites
*K*: Average number of differences
*H*_*d*_: Haplotype diversity
*π*: Nucleotide diversity
PCR: Polymerase chain reaction
*θ*_*0*_: Population sizes before expansion
*θ*_*1*_: Population size after expansion
SSD: Sum of squared deviations
HRI: Harpending’s raggedness index
AMOVA: Analysis of molecular variance
SAMOVA: Spatial analysis of molecular variance
τ: Age of expansion in units of mutational time
*F*_*CT*_: Variation among groups
*F*_*ST*_: Wright’s fixation index
*D**s*: Genetic distance
UPGMA: Unweighted pair group method with arithmetic means
NJ: Neighbor-joining
