# Supplementary material for: Genetic diversity and variation of seven Chinese grass shrimp (Palaemonetes sinensis) populations based on the mitochondrial COI gene
Source: BMC Ecol Evol. 2021 Sep 6;21:167. doi: 10.1186/s12862-021-01893-8 (PMC8422745; doi:10.1186/s12862-021-01893-8)
Supplement: Supplementary file 1 — Additional file 1: Table S1. Distribution of the COI haplotypes of P. sinensis. [file 12862_2021_1893_MOESM1_ESM.docx]

Table S1 Distribution of the *COI* haplotypes of *P. sinensis*

| Haplotype | Accession No. | Population | | | | | | | Sum |
| --- | --- | --- | --- | --- | --- | --- | --- | --- | --- |
|  |  | PJ | SL | AS | DL | SY | SH | JN |  |
| Hap_1 | MT884019 | 35 | 19 | 39 | 40 | 31 | 29 |  | 193 |
| Hap_2 | MT884020 | 1 |  |  |  |  |  |  | 1 |
| Hap_3 | MT884021 | 1 |  |  |  |  |  |  | 1 |
| Hap_4 | MT884022 | 1 |  |  |  |  |  |  | 1 |
| Hap_5 | MT884023 | 1 |  |  |  |  |  |  | 1 |
| Hap_6 | MT884024 | 1 |  |  |  | 1 |  |  | 2 |
| Hap_7 | MT884025 | 1 |  |  |  |  |  |  | 1 |
| Hap_8 | MT884026 | 1 | 10 | 1 |  |  | 8 |  | 20 |
| Hap_9 | MT884027 | 1 | 1 |  |  |  |  |  | 2 |
| Hap_10 | MT884028 | 1 |  |  |  |  |  |  | 1 |
| Hap_11 | MT884029 |  | 1 |  |  |  |  |  | 1 |
| Hap_12 | MT884030 |  | 1 |  |  |  |  |  | 1 |
| Hap_13 | MT884031 |  | 1 |  |  |  |  |  | 1 |
| Hap_14 | MT884032 |  | 1 |  |  |  |  |  | 1 |
| Hap_15 | MT884033 |  | 1 | 1 |  |  |  | 36 | 38 |
| Hap_16 | MT884034 |  | 1 |  |  |  |  |  | 1 |
| Hap_17 | MT884035 |  | 1 |  |  |  |  |  | 1 |
| Hap_18 | MT884036 |  | 1 |  |  | 11 |  |  | 12 |
| Hap_19 | MT884037 |  | 1 |  |  |  |  |  | 1 |
| Hap_20 | MT884038 |  | 5 |  | 2 |  | 4 |  | 11 |
| Hap_21 | MT884039 |  | 1 |  |  |  |  |  | 1 |
| Hap_22 | MT884040 |  | 2 |  |  |  |  |  | 2 |
| Hap_23 | MT884041 |  |  | 1 |  |  |  |  | 1 |
| Hap_24 | MT884042 |  |  | 4 |  |  | 1 |  | 5 |
| Hap_25 | MT884043 |  |  | 1 |  |  |  |  | 1 |
| Hap_26 | MT884044 |  |  |  | 2 |  |  |  | 2 |
| Hap_27 | MT884045 |  |  |  | 1 |  |  |  | 1 |
| Hap_28 | MT884046 |  |  |  | 1 |  |  |  | 1 |
| Hap_29 | MT884047 |  |  |  |  | 1 |  |  | 1 |
| Hap_30 | MT884048 |  |  |  |  | 1 |  |  | 1 |
| Hap_31 | MT884049 |  |  |  |  | 1 |  |  | 1 |
| Hap_32 | MT884050 |  |  |  |  | 1 |  |  | 1 |
| Hap_33 | MT884051 |  |  |  |  | 1 |  |  | 1 |
| Hap_34 | MT884052 |  |  |  |  |  | 1 |  | 1 |
| Hap_35 | MT884053 |  |  |  |  |  | 5 |  | 5 |
| Hap_36 | MT884054 |  |  |  |  |  |  | 1 | 1 |
| Hap_37 | MT884055 |  |  |  |  |  |  | 1 | 1 |
| Hap_38 | MT884056 |  |  |  |  |  |  | 5 | 5 |
| Hap_39 | MT884057 |  |  |  |  |  |  | 1 | 1 |
| Hap_40 | MT884058 |  |  |  |  |  |  | 1 | 1 |
| Hap_41 | MT884059 |  |  |  |  |  |  | 1 | 1 |
